# *NPM1* exon 5 mutations in acute myeloid leukemia: Implications in diagnosis and minimal residual monitoring

**DOI:** 10.1002/jha2.445

**Published:** 2022-04-26

**Authors:** Peng Wang, Jeremy Segal, Michael W. Drazer, Girish Venkataraman, Daniel A. Arber, Sandeep Gurbuxani

**Affiliations:** ^1^ Department of Pathology University of Chicago Chicago Illinois USA; ^2^ Department of Medicine University of Chicago Chicago Illinois USA

1

*Nucleophosmin 1* (*NPM1*) mutated acute myeloid leukemia is recognized as distinct entity in the 2016 revision of the WHO classification [1]. In patients with normal karyotype, presence of *NPM1* mutations results in improved risk from intermediate to favorable despite the presence of morphologic dysplasia [2, 3]. Monocytic morphology, blasts with cup like nuclear invaginations, and dim CD34 expression are all associated with *NPM1*‐mutated acute myeloid leukemia (AML); the findings are relatively nonspecific and definite diagnosis relies on demonstration of *NPM1* mutations that result in aberrant cytoplasmic localization of the NPM1 protein. The vast majority of *NPM1* mutations described affect exon 12 (NM_001355006 transcript, or exon 11 of the alternatively annotated transcript NM_002520). These mutations result in generation of an extra nuclear export signal in the C‐terminus of the NPM1 protein resulting in aberrant cytoplasmic localization [4]. Therefore, the vast majority of amplicon‐based assays currently used in clinical laboratories are designed to target the mutational hotspots in exon 12. However, emerging evidence suggests that other mutations besides the canonical exon 12 mutations may contribute to aberrant NPM1 cytoplasmic localization [5].

At our institution, somatic mutation analysis is routinely performed on all AML patients using a next‐generation sequencing with a large‐scale hybrid capture assay covers all exons of the *NPM1* gene [6]. In the context of emerging data regarding nonexon 12 *NPM1* mutations, we reviewed our clinical database of AML patients with *NPM1* variants to determine if additional cases with pathogenic *NPM1* mutations could be identified.

In the database of AML patients sequenced at our institution, canonical frameshift mutations were detected in *NPM1* exon 12 (NM_001355006), or exon 11 of the alternatively annotated transcript (NM_002520) in 92 patients. These included 89 patients with W288Cfs, and three patients with W290Cfs. An additional patient had a frameshift mutation F268Lfs*8, which would be expected to abrogate the nucleolar localization signal (NoLS) at NPM1 C‐terminus and to behave similarly to the other canonical mutations.

However, based on the review of data we identified two additional AML patients with *NPM1* exon 5 (NM_002520) mutations (Table 1). Patient 1 was a 73‐year‐old female with de novo AML and a 21‐bp in‐frame insertion at c.407_c.408, resulting in a 7‐amino acid insertion between Leu136 and Ser137 (Figure 1A). Patient 1 also had *BCOR*, *NRAS*, *PTPN11*, *RAD21* and *WT1* mutations. They were treated with azacitidine and venetoclax with complete hematopoietic recovery after cycle 1 of therapy. They continue on azacitdine and venetoclax with normal blood counts. Patient 2 was a 45‐year‐old male with de novo AML and a 21‐bp duplication of c.387_407, resulting in a duplication of the sequence between Glu130 and Leu136 (Figure 1B). Patient 2 also had *DNMT3A* and *IDH1* mutations. They were initially treated on the SWOG S1203 protocol [7] with idarubicin, cytarabine, and vorinostat with primary refractory disease. Second line of therapy was on protocol [8] with azacitidine, cytarabine, and mitoxantrone with residual disease followed by a hematopoietic stem and progenitor cell transplant before relapsing. They received two cycles of hypomethylating agent‐based therapies before dying of refractory disease. In both patients, the extra amino acids in the mutated *NPM1* generated an extra nuclear export signal (NES) motif ([9], Figure 1A,B). Both patients had normal karyotypes and no *FLT3* mutations. Similar to the cases described by Martelli and colleagues, bone marrow findings were notable for presence of dysplasia most notable in the megakaryocyte lineage (Figure 1C,D) and variable to absent CD34 expression. In order to determine the functional impact of the newly created nuclear export signal, we performed immunohistochemistry (IHC) using the "pan" NPM1 antibody (DAKO clone 376). IHC confirmed the aberrant NPM1 cytoplasmic localization (NPM1c+) (Figure 1E,F).

**TABLE 1:** Characteristics of two patients with *NPM1* exon 5 mutation

**Characteristics**	**Patient 1**	**Patient 2**
Gender	F	M
Age	73	45
Hb (g/dl)	7.4	8.4
PLT (k/μl)	45	106
WBC (k/μl)	2.9	1.3
Diagnosis	Acute myeloid leukemia	Acute myeloid leukemia
IHC	NPM1(c+)	NPM1(c+)
Karyotype	Normal	Normal
Dysplastic features	Multilineage, most notable in megakaryocytes	Multilineage, most notable in megakaryocytes
Blasts flow/CD34 expression	22%	24%
*FLT3*	WT	WT
*DNMT3A* (VAF%)	WT	*DNMT3A* p.R598* (7%) *DNMT3A* p.P385Rfs*22 (9%)
*IDH1/2*	WT	*IDH1* p.R132C (12%)
*BCOR*	p.V797Cfs*19 (26%)	WT
*NRAS*	p.G12A (15%)	WT
*PTPN11*	p.D61H (13%)	WT
*RAD21*	p.Q433* (17%)	WT
*WT1*	p.A387Vfs*4 (28%)	WT
First‐line therapy	Azacitidine, venetoclax (complete hematologic response)	Idarubicin 25 mg (days 4–6), cytarabine (3200 mg days 4–7), vorinostat (500 mg BID days 1‐3) (primary refractory disease)
Second‐line therapy	NA	Azacitidine, high‐dose cytarabine, mitoxantrone (residual disease)
Hematopoietic stem and progenitor cell conditioning	NA	Fludarabine, busulfan, alemtuzumab
Relapse #1 therapy (cycle 1)	NA	Azacitidine (progression)
Relapse #1 therapy (cycle 2)	NA	Decitabine (10 day), donor lymphocyte infusion

**FIGURE 1:**
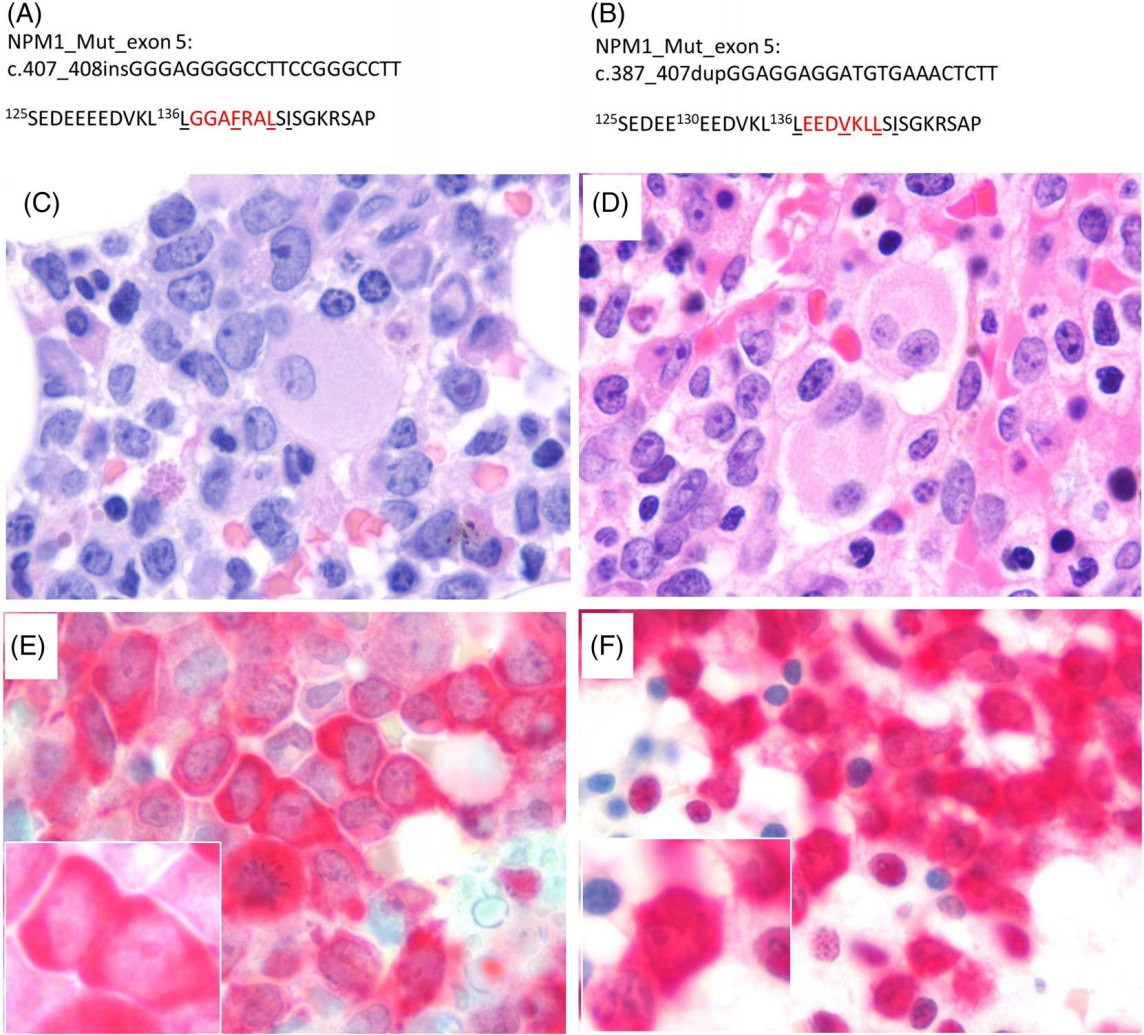
*NPM1* mutations in exon 5. (A, B) Predicted protein sequence of the exon 5 mutants in patients 1 and 2. Nucleotide insertions (patient 1) and duplication (patient 2) are shown according to the *NPM1* complementary DNA sequence. The newly acquired amino acids are highlighted in red and the predicted nuclear export signal motifs are underlined. (C, D) Hematoxylin and eosin‐stained bone marrow biopsies show extensive bone marrow involvement by leukemic blasts with folded nuclei and finely dispersed chromatin reminiscent of monocytic differentiation (total magnification 1000×). (E, F) Immunostaining performed with NPM1 antibody (Dako clone 376, pan NPM1) confirmed aberrant cytoplasmic as well as retained nucleolar staining (inset images) (total magnification 1000×)

Overall, we confirm that *NPM1* exon 5 in‐frame insertions or duplications are an uncommon but recurrent finding in AML. Because AML with mutated *NPM1* has been recognized as a distinct entity based on a single gene mutation by the WHO classification of AML [1], identification of *NPM1* mutations is critical for accurate AML diagnosis and patient management [10]. Both of our patients with mutations in *NPM1* exon 5 were re‐stratified from "Intermediate" to "Favorable" risk based on the 2017 European LeukemiaNet guidelines [2]. This revision in risk has direct implications for decisions regarding hematopoietic stem and progenitor cell transplant, and similar patients could be spared unnecessary upfront transplants in the future. Our observations further demonstrate the importance of either screening for nonexon 12 *NPM1* mutations with IHC, or whole *NPM1* gene sequencing to identify rare *NPM1* mutations outside of exon 12 for better classification of AML patients. Furthermore, similar to exon 12 mutations, exon 5 *NPM1* mutations can be followed either by RT‐PCR based assays [11] or highly sensitive error corrected next‐generation sequencing [12] to monitor measurable residual disease to obtain definitive information on the impact of *NPM1* exon 5 mutations in AML prognosis.

## FUNDING INFORMATION

None.

## CONFLICT OF INTEREST

The authors declare no conflict of interest.

## ETHICS STATEMENT

The study is deemed as exempt from IRB approval.

## AUTHOR CONTRIBUTION

Peng Wang and Sandeep Gurbuxani designed research, performed research, analyzed data, and wrote the paper; Michael W. Drazer, Girish Venkataraman, and Daniel A. Arber performed research and wrote the paper. Michael W. Drazer has served as a consultant to Cardinal Health.
